# Identification of TENP as the Gene Encoding Chicken Egg White Ovoglobulin G2 and Demonstration of Its High Genetic Variability in Chickens

**DOI:** 10.1371/journal.pone.0159571

**Published:** 2016-07-29

**Authors:** Keiji Kinoshita, Takeshi Shimogiri, Hisham R. Ibrahim, Masaoki Tsudzuki, Yoshizane Maeda, Yoichi Matsuda

**Affiliations:** 1 Avian Bioscience Research Center, Graduate School of Bioagricultural Sciences, Nagoya University, Nagoya, Japan; 2 Faculty of Agriculture, Kagoshima University, Kagoshima, Japan; 3 Laboratory of Animal Breeding and Genetics, Graduate School of Biosphere Science, Hiroshima University, Higashi-Hiroshima, Japan; 4 Laboratory of Animal Genetics, Department of Applied Molecular Biosciences, Graduate School of Bioagricultural Sciences, Nagoya University, Nagoya, Japan; Institute of Oceanology, Chinese Academy of Sciences, CHINA

## Abstract

Ovoglobulin G2 (G2) has long been known as a major protein constituent of chicken egg white. However, little is known about the biochemical properties and biological functions of G2 because the gene encoding G2 has not been identified. Therefore, the identification of the gene encoding G2 and an analysis of its genetic variability is an important step toward the goal of understanding the biological functions of the G2 protein and its utility in poultry production. To identify and characterize the gene encoding G2, we separated G2 from egg white using electrophoresis on a non-denaturing polyacrylamide gel. Two polymorphic forms of G2 protein (G2^A^ and G2^B^), with different mobilities (fast and slow respectively), were detected by staining. The protein band corresponding to G2^B^ was electro-eluted from the native gel, re-electrophoresed under denaturing conditions and its N-terminal sequence was determined by Edman degradation following transfer onto a membrane. Sequencing of the 47 kDa G2^B^ band revealed it to be identical to TENP (transiently expressed in neural precursors), also known as BPI fold-containing family B, member 2 (BPIFB2), a protein with strong homology to a bacterial permeability-increasing protein family (BPI) in mammals. Full-length chicken *TENP* cDNA sequences were determined for 78 individuals across 29 chicken breeds, lines, and populations, and consequently eleven non-synonymous substitutions were detected in the coding region. Of the eleven non-synonymous substitutions, A329G leading to Arg110Gln was completely associated with the noted differential electrophoretic mobility of G2. Specifically G2^B^, with a slower mobility is encoded by A329 (Arg110), whereas G2^A^, with a faster mobility, is encoded by G329 (Gln110). The sequence data, derived from the coding region, also revealed that the gene encoding G2 demonstrates significant genetic variability across different chicken breeds/lines/populations. These variants, and how they correlate with egg white properties, may allow us to understand further G2’s functions.

## Introduction

Avian egg albumen contains high levels of protein and is a major source of biologically active substances that are beneficial for human health. The physicochemical properties and functions of the major egg white proteins have been widely studied in the fields of food science, food biochemistry, and food processing for many years [1,2]. The primary role of the egg white is to protect both the yolk and the embryo from physical impact, and to supply developing embryos with physiologically active and anti-bacterial substances. At least forty different proteins are contained in egg white, and fourteen proteins are known to be major components of egg white, accounting for approximately 90–95% of total egg white proteins, including: ovalbumin, 54%; ovotransferrin, 12–13%; ovomucoid, 11%; lysozyme, 3.4–3.5%; ovoglobulin G3, 1.0–4.0%; ovoglobulin G2, 1.0–4.0%; ovomucin, 1.5–3.5%; ovoinhibitor, 0.1–1.5%; ovoflavoprotein (riboflavin binding protein), 0.8–1.0%; ovoglycoprotein, 0.5–1.0%; ovostatin, 0.5%; ovomacroglobulin, 0.5%; avidin, 0.05–0.5%; and cystatin, 0.01–0.05% [3,4,5,6], whereas, the remaining minor components are yet to be identified. More than one hundred proteins were found in egg white by proteomic analysis using two-dimensional gel electrophoresis (2DE) and mass spectrometry analysis [7,8]. However, it has remained unclear whether ovoglobulins have previously been included in the protein components identified by proteomic analysis. This is largely due to the lack of basic reference information such as monomeric molecular weight, isoelectric point (pI), and primary sequence.

Globulins are generally categorized as proteins that are sensitive to heat denaturation, soluble in a dilute salt solution, and are insoluble in water. The globulins found in egg white, ovoglobulins, are believed to be essential for the foaming property of egg white in food processing [9,10] and are principally classified into three types, G1, G2, and G3, based on electrophoretic mobility differences observed by moving-boundary electrophoresis [11]. G1 has been identified as lysozyme based on its abundance in egg white, its isoelectric point, and its electrophoretic mobility [12]. However, G2 and G3 remain to be characterized. The problem is somewhat confounded by the fact that there are significant variations in both molecular weights and total egg white abundance reported by a number of previous studies [13,14,15,16,17]. Furthermore the primary sequences of G2 and G3 have not been determined. G2 is currently assumed to account for 1% to 4% of total egg white protein, with a reported monomeric molecular weight ranging from approximately 30 kDa to 49 kDa [4,6,13,14]. Electrophoretic polymorphism of G2 in egg white has been surveyed by using non-denaturing polyacrylamide or starch gel electrophoresis since the 1960s, and two principal polymorphic forms (G2^A^ and G2^B^) with a difference in electrophoretic mobility are found in domestic chickens and red jungle fowl (*Gallus gallus*) [18,19,20,21,22]. Peptide mapping, using Cleveland gels, revealed that G2^A^ and G2^B^ differ in their sensitivity to V8 protease and chymotrypsin, and that G2^A^ is more acidic than G2^B^ based on its isoelectric point [16]. However, the difference in nucleotide sequence between these two *G2* alleles is currently unknown because the primary sequence of G2 has not been determined.

In this study, we isolated the G2 protein electrophoretically and determined its N-terminal amino acid sequence by Edman degradation. We then determined the nucleotide sequence of the cDNA encoding G2 and identified causative mutations leading to the previously noted electrophoretic mobility variants. We also assessed genetic polymorphisms of the G2 gene using a variety of chicken breeds, lines, and populations, as a means to evaluate the genetic diversity of the gene in chickens. Here, we report that egg white G2 is identical to the TENP (transiently expressed in neural precursors) protein, also known as BPI-fold containing family B, member 2 (BPIFB2) whose expression has been detected in chicken embryos and the chicken oviduct, and furthermore that the two electrophoretic forms of G2 are attributable to a single amino acid substitution in TENP.

## Materials and Methods

### Ethics statement

Animal care and all experimental procedures were approved by the Animal Experiment Committee, Graduate School of Bio-agricultural Sciences, Nagoya University (approval no 2014021202), and the experiments were conducted according to the ‘Regulations on Animal Experiments at Nagoya University’.

### Electrophoresis of the G2 Protein and Detection of Polymorphisms

In total, 285 egg white samples were collected from 27 chicken breeds, lines, and populations including a closed colony of red jungle fowl (Table 1). These samples were subjected to electrophoresis on non-denaturing polyacrylamide gels (38 x 38 x 1 mm) according to the method of Davis [23]. Briefly, thin egg white samples were diluted with 4 volumes of dilution buffer (2 ml of 0.5 M Tris-HCl [pH 6.8], 1.6 ml glycerol, and 0.4 ml of 0.05% [wt/vol] bromophenol blue), and electrophoresis was performed at 4°C at a constant current of 20 mA for 30min on a 4.5% stacking gel and at 50 mA for 3.5h on an 8% separating gel with 25 mM Tris-192 mM glycine buffer (pH 8.3). The separating gel was stained with 0.125% (wt/vol) Coomassie brilliant blue R-350 (CBB-R350) in methanol:acetic acid:water (40:7:53), and the protein bands were detected following destaining in methanol:acetic acid:water (25:10:65). Polymorphic differences in G2 were detected by assessing the presence of differential migrating protein bands corresponding to G2.

**Table 1:** Distribution of ovoglobulin G2 genotypes in 27 chicken breeds, lines, and populations surveyed in this study.

	Number of hens	Electrophoretic genotype			Frequency of *G2* allele	
Breed/line/population		*G2*^*B*^/*G2*^*B*^	*G2*^*A*^/*G2*^*B*^	*G2*^*A*^/*G2*^*A*^	*G2*^*A*^	*G2*^*B*^
Ehime-Jidori (EJ)	82	55	23	4	0.19	0.81
Chabo (Japanese Bantam) (JB)	12	6	6		0.25	0.75
Chahn (CHN)	12		1	11	0.96	0.04
White egg layer	20	8	11	1	0.32	0.68
Brown egg layer	20	6	14		0.35	0.65
Brown Leghorn (BL-E)	8	8				1.00
Black Minorca (BM-C)	8	8				1.00
White Leghorn (WL-G)	8	8				1.00
White Leghorn (WL-M/O)	8	8				1.00
White Leghorn (WL-JL)	8	8				1.00
White Leghorn (OS)	8	8				1.00
Fayoumi (GSP)	8	8				1.00
Fayoumi (PNP/DO)	8	8				1.00
Fayoumi (GSN/1)	8	8				1.00
Fayoumi (YL)	8	8				1.00
Polish Bantam (PB)	8	8				1.00
Cochin Bantam (CB)	2	2				1.00
Mille Fleur Bantam (MIL)	2	2				1.00
Modern Game Bantam (GB)	2	2				1.00
Australorp (AL-NU)	8	8				1.00
Rhode Island Red (RIR-Y8/NU)	8	8				1.00
Rhode Island Red (sex-linked dwarfism)	2	2				1.00
New Hampshare (413)	4	4				1.00
Koshamo (KOS)	8	8				1.00
Ukkokei (SIL)	8			8	1.00	
Nagoya	3	3				1.00
Red Jungle Fowl (RJF/NU)	4	4				1.00

### Extraction of G2 Proteins from Gels

Fresh thin egg white was collected from two hens in Ehime-Jidori, which exhibited the two different electrophoretic forms of G2 (G2^A^ and G2^B^). G2 bands were visualized by native-PAGE analysis as described above. The G2 bands were excised from the gel and extracted with 25 mM Tris-192 mM glycine buffer (pH 8.3) at 4°C using an electroeluter (Bio-Rad Laboratories, Hercules, CA, USA). The eluted samples were desalted using dialysis against distilled deionized water for 16 h at 4°C. The samples were subsequently lyophilized and stored at −80°C until analysis.

### SDS-PAGE and the N-terminal Amino Acid Sequencing of G2 Protein

The G2^B^ form sample of the G2 protein extracted from the gel was mixed with an equal volume of SDS sample buffer (2 ml of 0.5 M Tris-HCl [pH 6.8], 1.6 ml glycerol, 3.2 ml of 10% [wt/vol] SDS, 0.8 ml of β-mercaptoethanol, and 0.4 ml of 0.05% [wt/vol] bromophenol blue). Electrophoresis on 10% SDS-PAGE gels was performed according to the method of Laemmli [24], and the gel was stained and then destained using the same method, as described for native gel electrophoresis. The G2^B^ protein on the gel was electroblotted onto a polyvinylidene difluoride (PVDF) membrane, using a semi-dry blotter system at a constant current of 20 mA for 1.5 h as described by Towbin *et al* [25]. After transfer, the membrane was stained with 0.1% CBB-R350, 40% methanol and 1% acetic acid for a few minutes and destained in 50% methanol until the bands became clearly visible. The G2^B^ protein on the PVDF membrane was placed directly into a Model 492 Procise^®^ cLC capillary protein sequencer (Applied Biosystems, Carlsbad, CA, USA) for automatic Edman degradation analysis according to the manufacture’s protocol. Partial amino acid sequence data obtained by the N-terminal protein sequencing was used to search for homologous proteins with a translated BLAST search (tblastn) on the NCBI BLAST homepage (http://www.ncbi.nlm.nih.gov/BLAST/) [26].

### RT-PCR and Nucleotide Sequencing of G2 cDNAs from Different Chicken Sources

In total, 78 individuals, consisting of 21 egg-laying hens and 57 embryos, were used to sequence the cDNAs encoding the respective G2 protein (S1 Table). Animals were sacrificed by cervical dislocation, and a small piece of the oviduct magnum tissue was taken from each animal and quickly placed in RNAlater (Ambion, Austin, TX, USA). Whole embryos, lacking the head, were taken from five-day post incubation eggs and placed in RNAlater. Total RNA was extracted from these samples using TRIzol reagent according to the manufacturer's instruction (Invitrogen, Carlsbad, CA, USA). The quality of RNA was evaluated electrophoretically on a 2% agarose gel. Total RNA (1.5 μg) was reverse transcribed using a PrimeScript RT-PCR Kit (Takara, Otsu, Japan) according to the manufacturer's instruction. The PCR reaction mixture contained 20‒50 ng of cDNA, 1 x KOD FX buffer, 200 μM of each dNTP, 1.5 μl of each of forward and reverse primers (10 μM each), and 1 unit of KOD FX DNA polymerase (TOYOBO, Osaka, Japan) in a final volume of 50 μl. The PCR reaction was performed on a GeneAmp PCR system 9700 (Applied Biosystems) with the following cycle: initial denaturation for 2 min at 94°C, 35 cycles of 98°C for 10 s, 65°C for 30 s, and 68°C for 1.5 min, and final extension at 72°C for 7 min. The PCR products were purified from the gel using a Gel-M gel extraction kit (Viogene, Umeå, Sweden), and nucleotide sequences were determined using an ABI PRISM3130 DNA Analyzer after completion of a sequencing reaction using a Big Dye Terminator Cycle Sequencing Kit v3.1 (Life Technologies-Applied Biosystems). Sequence data sets were analyzed using ATGC sequence assembly software (Ver.5) (Genetyx, Tokyo, Japan).

### Genotyping of the G2 Gene

Genotyping of the gene encoding G2 was performed for a wide variety of chicken breeds, lines, and populations using a PCR-RFLP (restriction fragment length polymorphism) method (detailed below). Whole blood samples (1–5 ml) were collected from the wing vein of each bird using heparinized syringes, and genomic DNA was extracted from 10 μl of whole blood using the DNAZOL BD reagent (Molecular Research Center, Inc., Cincinnati, OH, USA). PCR products were digested with restriction endonucleases, electrophoresed on a 2% agarose gel, and the resultant DNA bands were visualized by staining with ethidium bromide.

## Results

### Isolation of Ovoglobulin G2 Variants

**Fig 1:**
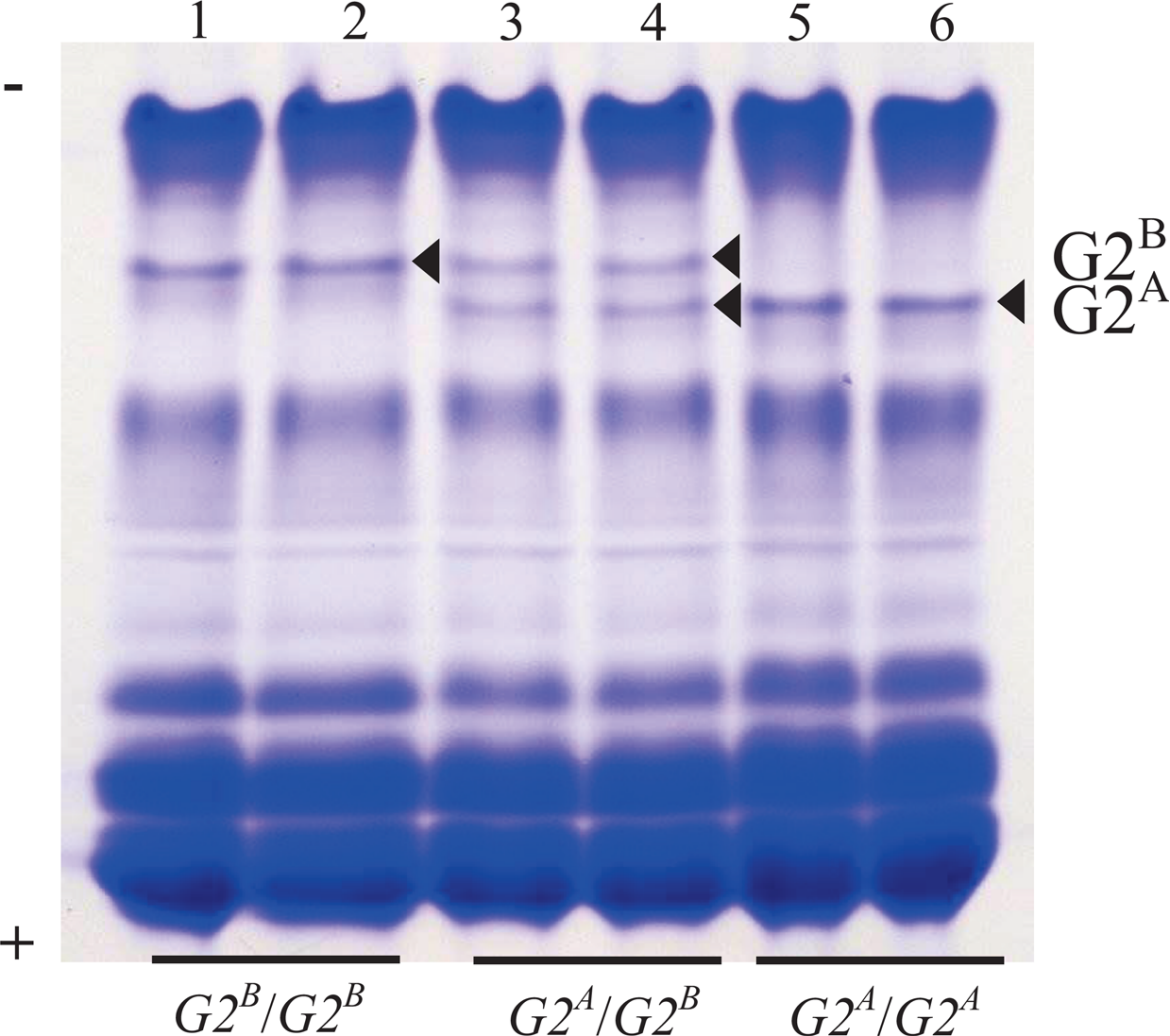
Representative Native Gel Electrophoretic Patterns of Chicken Egg White Proteins. Total chicken egg white proteins were separated by electrophoresis on an 8% non-denaturing polyacrylamide gel and stained with Coomassie blue. Arrowheads indicate the stained ovoglobulin G2 bands (G2^A^ and G2^B^). Lane 1–2, *G2*^*B*^/*G2*^*B*^ phenotype in Ehime-Jidori; lane 3–4, *G2*^*A*^/*G2*^*B*^ phenotype in Ehime-Jidori; lane 5–6, *G2*^*A*^/*G2*^*A*^ phenotype in Ehime-Jidori

Electrophoretic polymorphisms in egg white G2 protein were surveyed for 27 chicken breeds, lines, and populations (Table 1). Two G2 protein bands with differing mobility were detected in egg white (namely, G2^A^ and G2^B^) by native-PAGE (Fig 1): G2^A^ had a faster mobility than G2^B^. Egg white homozygous for either G2^A^ or G2^B^ showed a single G2 band of the appropriate electrophoretic mobility whereas egg white heterozygous for G2 showed both G2 bands. Of the 27 populations used in this study, 21, including red jungle fowl (RJF/NU), were monomorphic at the G2 loci having a phenotype *G2*^*B*^/*G2*^*B*^. Ukkokei (SIL) was found to be the only population with a *G2*^*A*^/*G2*^*A*^ phenotype, and the remaining five populations including Ehime-Jidori (EJ) were polymorphic (Table 1).

**Fig 2:**
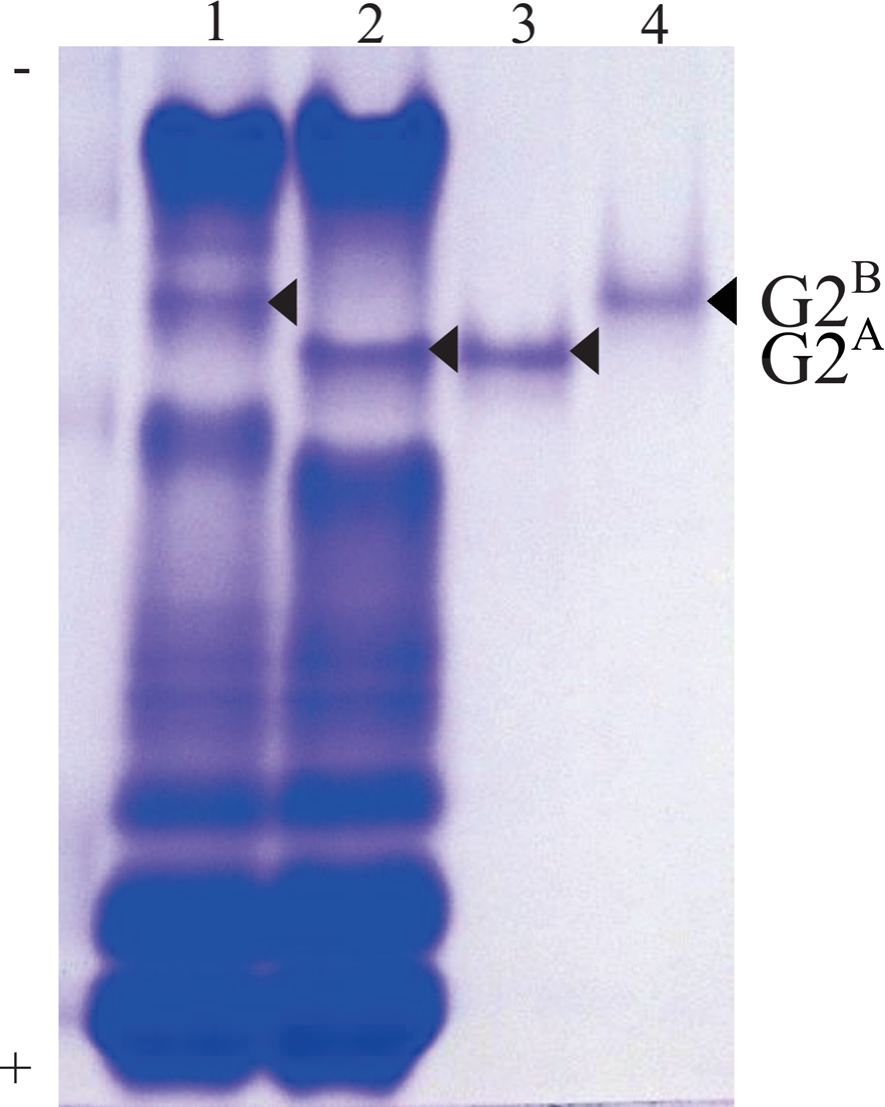
Native Gel Electrophoresis of Chicken Egg White Proteins Compared to Gel-eluted Samples of Ovoglobulin G2^A^ and G2^B^. Total chicken egg white proteins were separated by electrophoresis on an 8% non-denaturing polyacrylamide gel and stained with Coomassie blue. Arrowheads indicate the stained ovoglobulin G2 bands (G2^A^ and G2^B^). Lane 1–2, G2^B^/G2^B^ band (lane 1) and G2^A^/G2^A^ band (lane 2) in total egg white extracts. Lane 3–4, gel-eluted ovoglobulin G2 from G2^A^/G2^A^ band (lane 3) and G2^B^/G2^B^ band (lane 4).

**Fig 3:**
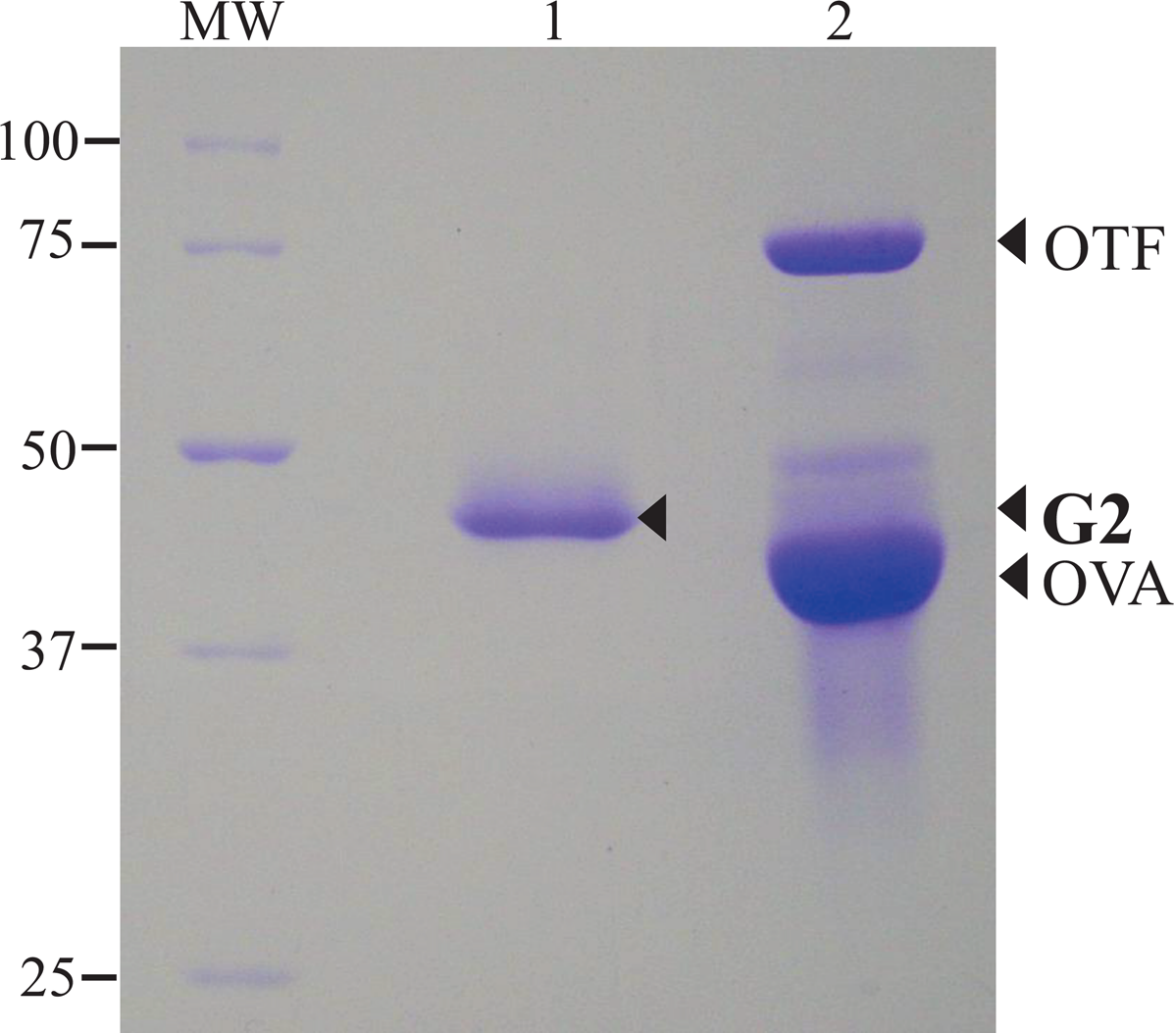
Denaturing Gel Electrophoresis (10% SDS-PAGE) of Gel-eluted Ovoglobulin G2^B^ Compared to Purified Ovotransferrin and Ovalbumin. Lane 1, Ovoglobulin G2^B^/G2^B^ electroeluted from a native PAGE gel. Lane 2, a mixture of purified egg white ovotransferrin (OTF) and ovalbumin (OVA). MW, Precision Plus Protein Standards (BIO-RAD, Hercules, CA).

EJ chickens were selected as a source of G2 because they have both G2^A^ and G2^B^ and they have been maintained as a closed population. The two G2 forms were identified based on their differing electrophoretic mobility on 8% native-gels under non-denaturing conditions (Fig 2 lanes 1,2) with G2^A^ having the faster electrophoretic mobility. The two forms were electroeluted from the gel, and the eluted samples were confirmed to be a single band by re-electrophoresis using 8% native-PAGE (Fig 2; lane 3, 4). The monomeric molecular mass of G2 was estimated to be approximately 47 kDa using 10% SDS-PAGE (Fig 3).

### N-terminal Amino Acid Sequence of G2

We excised the G2^B^ protein band and used it to determine the N-terminal amino acids of the G2 protein by Edman degradation. The first fifteen N-terminal amino acids of G2^B^ protein were TRAPDCGGILTPLGL. The purity of the samples was confirmed by the absence of significant background interference during sequencing. A tblastn search revealed that the N-terminal amino acid sequence of G2^B^was identical to the amino acid resides 14–28 of chicken TENP (transiently expressed in neural precursors) (Accession no. AF029841), so named, because it is transiently expressed prior to overt cell differentiation of neural precursor cells during neurogenesis in chicken embryos [27]. The N-terminal sequence of the G2 protein determined in this study did not contain the first 13 N-terminal amino acid residues (MGALLALLDPVQP) of TENP, likely because this sequence is predicted as a putative processed signal peptide by SOSUIsignal (http://bp.nuap.nagoya-u.ac.jp/sosui/sosuisignal/) [28]. The signal cleavage site of G2^B^ was completely identical to that of the chicken TENP (Accession no. HG007958) proposed by Whenham *et al* [29]. These results suggest that the egg white G2^B^ protein is the product of the *TENP* gene.

### Mutations Causative for G2 Electrophoretic Variants

**Fig 4:**
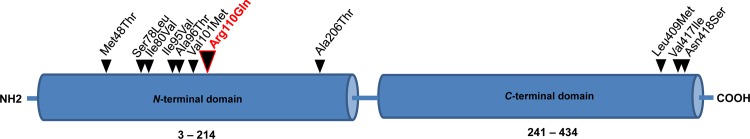
Schematic Representation of Eleven Amino Acid Substitutions and their Corresponding Positions in TENP. One amino acid substitution (Ala206Thr), which is associated with the electrophoretic mobility difference between the G2^A^ and G2^B^ forms, is shown in red.

To identify the presumptive mutation underlying the differences in the electrophoretic mobility of G2 proteins, we determined the nucleotide sequences of full length *TENP* cDNAs prepared from the oviduct RNA from 21 egg-laying hens as shown in S1 Table. In addition, the full-length cDNA sequences of *TENP* from 57 embryos representing 23 breeds, lines, and populations were also determined. RT-PCR was performed with a pair of primers (Tenp_F1 and Tenp_R1) and internal primers were used for direct sequencing with *TENP* cDNA as template (Table 2). These primers were designed based on the nucleotide sequence of the chicken *TENP* gene (AF029841). Comparisons of the *TENP* cDNA sequences among 21 hens and 57 embryos identified a total of 21 SNPs, including 10 synonymous substitutions at positions 312, 426, 594, 807, 843, 846, 870, 897, 1170, and 1201, and, 11 non-synonymous substitutions at positions 143, 233, 238, 283, 286, 301, 329, 616, 1225, 1249, and 1253 (S1 Table, Table 3, Fig 4). Eleven non-synonymous substitutions found including Thr48Met, Ser78Leu, Ile80Val, Val95Ile, Thr96Ala, Val101Met, Arg110Gln, Ala206Thr, Met409Leu, Val417Ile, and Ser418Asn. The sequences obtained in this study were deposited in the DNA Data Bank of Japan (DDBJ; http://www.ddbj.nig.ac.jp/index-e.html; accession numbers, AB219157 –AB219159, LC144559 –LC144608).

**Table 2:** List of primers used for amplification and sequencing of the chicken *TENP* gene.

Primer name	Location	Nucleotide sequence (5′ to 3′)	Nucleotide position on the coding DNA reference sequence^a^	Nucleotide position on the reference sequence^b^
Tenp_F1	5’UTR	AGCCGGGAGGATGGGAACAGCAAAC	‐	10647531‒10647555
Tenp_R1	3’UTR	TGCGGTGAAAATCCACAAAGCTGAGCACTG	1424‒1396	10643103‒10643132
Tenp_ex3RS^c^	exon 3	GGTCAGCCACTTTTACAG	269‒253	10646576‒10646593
Tenp_ex5FS^c^	exon 5	CAAAGTGGTGGATGTCGA	502‒520	10645813‒10645830
Tenp_ex8RS^c^	exon 8	ATGGGATGTGCTGGTGCT	769‒753	10645122‒10645139
Tenp_ex10FS^c^	exon 10	TCACTGTTCACATTGGGG	1002‒1020	10644625‒10644642
Tenp_ex14FS^c^	exon 14	GTCCCACTTCACCTACAC	1276‒1294	10643440‒10643457
Tenp_ex3F^d^	exon 3	ATCGAGGTGGAGCTGCGCGTCG	311‒333	10646513‒10646534
Tenp_int4R^d^	intron 4	AGCTCTTGGTCCTTCCACCTTCCTG	‐	10646180‒10646204
Tenp_int6F^d^	intron 6	TTCCAGGCACCAGAAATCTGGGAG	‐	10645575‒10645598
Tenp_ex8R^d^	exon 8	TAGAAGTGCTCGGACAAAGCCAGG	798‒781	10645093‒10645116

^a^ cDNA sequence of *Gallus gallus TENP* gene (accession no. AF029841).

^b^ Reference sequence of chicken chromosome 20 (NC_006107) taken from the genome assembly Gallus_gallus-4.0/galGal4 (http://www.ncbi.nlm.nih.gov/assembly/GCF_000002315.3/).

^c^ Primers used only for DNA sequencing.

^d^ Primers used for PCR-RFLP analysis and partial genomic DNA sequencing.

**Table 3:** Nucleotide and deduced amino acid substitutions in the chicken *TENP* gene detected in this study.

Exon	SNP (position)^a^	Amino acid (position)^a^
2	**C>T (143)**	**Thr>Met (48)**
3	**C>T (233)**	**Ser>Leu (78)**
3	**A>G (238)**	**Ile>Val (80)**
3	**G>A (283)**	**Val>Ile (95)**
3	**A>G (286)**	**Thr>Ala (96)**
4	**G>A (301)**	**Val>Met (101)**
4	G>A (312)	Pro (104)
4	**G>A (329)**	**Arg>Gln (110)**
4	A>G (426)	Ala (142)
7	C>T (594)	Pro (198)
7	**G>A (616)**	**Ala>Thr (206)**
8	T>C(807)	Pro (269)
9	C>T (843)	Ile (281)
9	T>C (846)	Thr (282)
10	A>G (870)	Glu (290)
10	G>A (897)	Ala (299)
13	G>A (1170)	Gln (390)
14	C>A (1201)	Arg (401)
14	**A>C (1225)**	**Met>Leu (409)**
14	**G>A (1249)**	**Val>Ile (417)**
14	**G>A (1253)**	**Ser>Asn (418)**

Bold letters indicate non-synonymous substitutions.

^a^ The *TENP* cDNA sequence of the red jungle fowl (DDBJ accession no. LC144603) was used as a wild-type reference for the position of nucleotides and deduced amino acids and their substitutions.

We examined the association of non-synonymous substitutions in the *TENP* gene with electrophoretic mobility differences in the G2 protein. The results showed that two of the eleven non-synonymous substitutions, G329A in exon 4 (Arg110Gln) and G616A in exon 7 (Ala206Thr), corresponded to the egg white G2 phenotype; A329 (Gln110) and A616 (Thr206) were from the *G2*^*A*^ allele and G329 (Arg110) and G616 (Ala206) were from the *G2*^*B*^ allele in at least 21 hens.

An N-linked glycosylation site is predicted at Asn265 in the G2 protein (see NetNglyc 1.0 Server (http://www.cbs.dtu.dk/services/NetNGlyc/) [30]). However, there were no amino acid substitutions noted at this position, indicating that N-linked glycosylation is, most likely, not associated with the differences in G2 electrophoretic mobility.

### Association between Nucleotide Substitutions in *TENP* and Electrophoretic Polymorphism in G2

To confirm the association between the two non-synonymous substitutions, G329A and G616A, from the *TENP* gene and the electrophoretic mobility difference in egg white G2, nucleotide sequences at the two substitution sites and electrophoretic mobility were examined for 412 individuals from twenty six different chicken breeds, lines, and populations, including a colony of red jungle fowl, as shown in Table 4. A 355-bp fragment including exon 4 and a 506-bp fragment including exon 7 were amplified separately using Tenp_ex3F/Tenp_int4R primers and with Tenp_int6F/Tenp_ex8R primers, respectively (Table 2) using genomic DNA as a template. The primers used for amplification and sequencing were designed based on a reference sequence from chicken chromosome 20 (NC_006107) taken from the genome assembly Gallus_gallus-4.0/galGal4 (http://www.ncbi.nlm.nih.gov/assembly/GCF_000002315.3/). The 355-bp and 506-bp PCR products were digested with *Bcn*I and *Hph*I restriction enzymes, respectively, andelectrophoresed.

**Table 4:** Association between ovoglobulin G2 phenotypes and two non-synonymous substitutions (G329A and G616A) of the *TENP* gene in 26 chicken breeds, lines, and populations.

	Number of birds		Nucleotide substitution (Amino acid substitution)					
Breed/line/population		G2 phenotype	G329A (Arg110Gln)			G616A//(Ala206Thr)		
			G/G	G/A	A/A	G/G	G/A	A/A
Ehime-Jidori (EJ)	8	AA			8			8
	25	AB		25			25	
	40	BB	40			40		
Chabo (Japanese Bantam) (JB)	4	AA			4			4
	26	AB		26			26	
	30	BB	30			30		
Chahn (CHN)	22	AA			22			22
	7	AB		7			7	
White egg layer	1	AA			1			1
	4	AB		4			4	
	3	BB	3			3		
Brown egg layer	8	AB		8			8	
	1	BB	1			1		
Brown Leghorn (BL-E)	8	BB	8			8		
Black Minorca (BM-C)	8	BB	8					8
White Leghorn (WL-G)	8	BB	8			8		
White Leghorn (WL-M/O)	8	BB	8			8		
White Leghorn (WL-JL)	12	BB	12			12		
White Leghorn (OS)	10	BB	10			10		
Fayoumi (GSP)	8	BB	8			8		
Fayoumi (PNP/DO)	8	BB	8			8		
Fayoumi (GSN/1)	8	BB	8			8		
Fayoumi (YL)	8	BB	8			8		
Polish Bantam (PB)	46	BB	46			46		
Cochin Bantam (CB)	6	BB	6			6		
Mille Fleur Bantam (MIL)	6	BB	6			6		
Modern Game Bantam (GB)	6	BB	6			6		
Australorp (AL-NU)	8	BB	8			8		
Rhode Island Red (RIR-Y8/NU)	8	BB	8			8		
Rhode Island Red (sex-linked dwarf)	5	BB	5			5		
New Hampshare (413)	8	BB	8					8
Koshamo (KOS)	40	BB	40			40		
Ukkokei (SIL)	10	AA			10			10
Red Jungle Fowl (RJF/NU)	4	BB	4			4		

**Fig 5:**
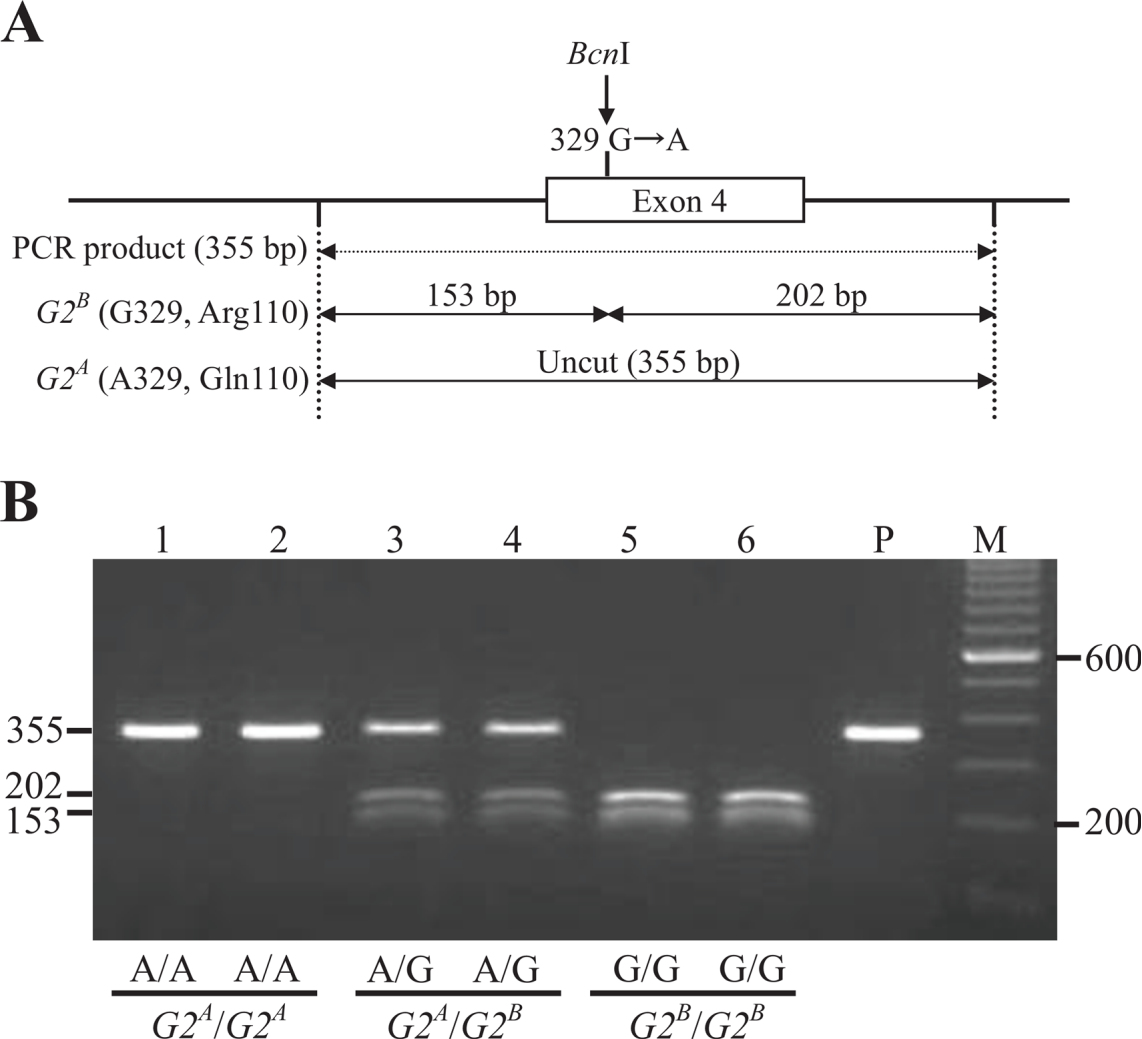
Genotyping of G329A Substitution in the *TENP* Gene by the PCR-RFLP Method and Comparison of the Genotypes with *G2*^*A*^ and *G2*^*B*^ alleles. (A) Schematic representation of the *Bcn*I-RFLP in the 355-bp PCR product amplified from *TENP* exon 4 using genomic DNA as a template. Digestion of the PCR product with *Bcn*I (5′-CC↓SGG-3′ or 3′-GGS↑CC-5′) is predicted to produce two DNA fragments (153 bp and 202 bp) in the case of the *G2*^*B*^ allele, whereas the PCR product derived from the *G2*^*A*^ allele is predicted to be not digested with *Bcn*I. (B) Genotyping using 2% agarose gel electrophoresis of the PCR products digested with *Bcn*I. The nucleotides at position 329 and genotypes of ovoglobulin G2 in six individuals are indicated below the lanes. Lane 1‒2, A/A (*G2*^*A*^/*G2*^*A*^); lane 3‒4, A/G (*G2*^*A*^/*G2*^*B*^); lane 5‒6, G/G (*G2*^*B*^/*G2*^*B*^); P, undigested PCR product; M, Ready-Load 100 bp DNA Ladder (0.1‒2 kb) (Invitrogen, Carlsbad, CA, USA).

**Fig 6:**
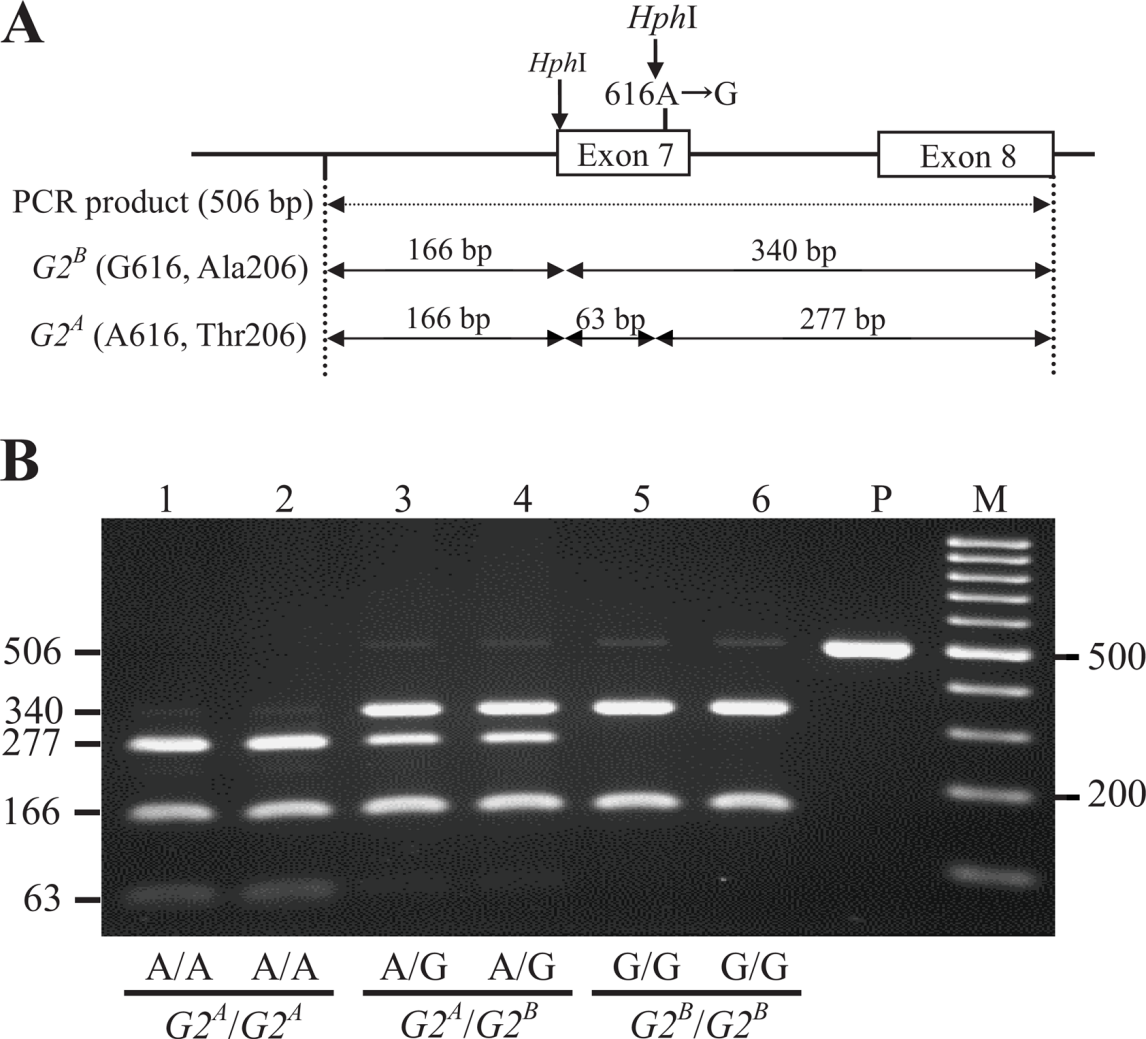
Genotyping of the G616A Substitution in the *TENP* Gene by the PCR-RFLP Method and Comparison of the Genotypes with *G2*^*A*^ and *G2*^*B*^ alleles. (A) Schematic representation of the *Hph*I-RFLP in the 506-bp PCR product amplified from genomic DNA (encompassing exon 7 and exon 8 of the *TENP* gene). Digestion with *Hph*I (5′-GGTGA(N)_8_↓-3′ or 3′-CCACT(N)_7_↑-5′) is predicted to produce three DNA fragments (166 bp, 63 bp, and 277 bp) in the *G2*^*A*^ allele, whereas two fragments are predicted (166 bp and 340 bp) from the *G2*^*B*^ allele. (B) Genotyping using 2% agarose gel electrophoresis of the PCR products digested with *Hph*I. The nucleotides at position 616 and genotypes of ovoglobulin G2 are shown at the bottom of the lanes. Lane 1‒2, A/A (*G2*^*A*^/*G2*^*A*^) in Ehime-Jidori; lane 3‒4, A/G (*G2*^*A*^/*G2*^*B*^) in Ehime-Jidori; lane 5‒6, G/G (*G2*^*B*^/*G2*^*B*^) in Ehime-Jidori; P, undigested PCR product; M, 100 bp DNA Ladder RTU (0.1‒3 kb) (NIPPON Genetics, Tokyo, Japan).

The G329A mutation in exon 4 of *TENP* destroyed the *Bcn*I recognition site (5′-CC↓SGG-3′ or 3′-GGS↑CC-5′, Fig 5A). Two DNA fragments (153 bp and 202 bp) were produced from the *G2*^*B*^ allele by *Bcn*I digestion of the 355 bp PCR fragment, whereas the *G2*^*A*^ allele showed an undigested single DNA band (355 bp, Fig 5B). The G616A mutation in exon 7 of *TENP* caused the disappearance of one of two *Hph*I restriction site (5′-GGTGA(N)8↓-3′ or 3′-CCACT(N)7↑-5′) in the 506 bp fragment (Fig 6A), and, therefore, 3 DNA fragments (166 bp, 63 bp, and 277 bp, respectively) were produced from the *G2*^*A*^ allele by *Hph*I digestion, whereas two DNA fragments (166 bp and 340 bp) were produced from the *G2*^*B*^ allele (Fig 6B). In all of the 412 individuals, A329 (Gln110) and A616 (Thr206) were highly associated with the *G2*^*A*^allele, and G329 (Arg110) and G616 (Ala206) with the *G2*^*B*^ allele (Table 4). However, two exceptions were found, namely for Black Minorca (BM-C) and New Hampshire (413) lines having the phenotype G2^B^/G2^B^; these lines possessed Arg at position 110 and Thr at position 206. Overall these results predict that only G329A SNP (Arg110Gln) is responsible for the difference in electrophoretic mobility between the *G2*^*A*^ and *G2*^*B*^ alleles.

## Discussion

Previously, G2 was fractionated from chicken egg white using basic protein isolation methods such as ammonium sulfate precipitation and/or chromatographic separation, and basic information, such as molecular weights and isoelectric points (pI) were characterized [13,14,16]. Additionally, more than 100 egg white protein components were identified by proteomics analysis [7,8], however, the G2 protein remained unidentified. This was because its primary amino acid sequence was unknown [13,14,15,16,17]. G2 can be easily separated from other egg white proteins using starch and/or non-denaturing acrylamide gel electrophoresis. Two principal polymorphic forms (G2^A^ and G2^B^) having different electrophoretic mobilities are found in variety of different chicken populations [18,19,20,22,31,32,33,34]. Electrophoretic separation is, therefore, a convenient and reliable means for separating the G2 protein from other egg white proteins. Further, the presence of two polymorphic forms is useful for confirming whether *G2* and a putative candidate gene are the same. In this study, we separated the polymorphic forms of the G2 protein as single protein bands following electrophoresis of egg white proteins using native-PAGE. The two polymorphic forms (G2^A^ and G2^B^) were electroeluted directly from the gel, and when re-electrophoresed under denaturing conditions by SDS-PAGE both the forms had a molecular weight of 47 kDa, which compared favorably to that reported in previous studies (49 kDa, Nakamura *et al* [14]; 47 kDa, Stevens and Duncan [16]); however, it was substantially different from that reported by Feeny (35 kDa) [13]. The first fifteen N-terminal amino acid residues (TRAPDCGGILTPLGL) of the G2 protein corresponded completely to those at position 14 to 28 of TENP (AF029841), reported by Yan and Wang [27]. The SOSUIsignal (http://bp.nuap.nagoya-u.ac.jp/sosui/sosuisignal/) predicted that the first 13 N-terminal amino acid residues (MGALLALLDPVQP) of TENP (AF029841) correspond to a signal peptide. This putative G2 signal cleavage site was identical to that of chicken TENP (HG007958) proposed by Whenham *et al* [29], and the N-terminal amino acid residues of the chicken G2 protein were also homologous to those of the emu (*Dromaius novaehollandiae*) TENP (AB556937) [35]. The theoretical molecular weight and pI values of the two TENP forms (426 amino acids), which excluded the first 13 amino acid residues, were expected to be 47.4 kDa and 5.67, respectively, for the G2^B^ allele (AB219158, Kinoshita submitted to GenBank, 23 Jun, 2005) and 47.4 kDa and 5.56, respectively, for the G2^A^ allele (AB219157, Kinoshita 2005) (Expasy compute pI/MW tool (http://web.expasy.org/compute_pi/))). These predicted molecular mass values correspond favorably to those obtained by SDS-PAGE electrophoresis of the native G2 protein (47 kDa). Our results, therefore, provide strong evidence that the egg white ovoglobulin G2 and the protein product of the *TENP* gene found in chicken embryos are identical proteins encoded by the same gene.

Chicken TENP was first identified as a nearly embryonic protein, which was transiently expressed in neural precursor cells in retina and brain [27]. Subsequently, it was also identified as a component of egg white, vitelline membrane, eggshell, and egg yolk in chickens by proteomic analysis [7,8,36,37,38]. The chicken *TENP* gene consists of 16 exons encoding 439 amino acids and is located at position 10,642,930−10,647,543 on chicken chromosome 20 (NC_006107.3). TENP is a member of the BPI (bactericidal/permeability-increasing protein) fold-containing family B (BPIFB) [39], and chicken TENP was classified as BPIFB7 (BPI fold-containing family B member 7) as a new family gene [39,40]. Chicken BPIFB7 shows the highest homology with mammalian BPIFB2 (BPI fold-containing family B member 2) (also known as BPIL1, BPI-increasing protein-like 1), which is highly expressed in the tonsils in *Homo sapiens* [41]. Therefore, chicken TENP is now officially named BPIFB2 (NCBI gene ID 395882). The human BPIFB2 is an antibacterial and endotoxin-neutralizing protein and is a key component of the innate immune system involved in defense against bacteria. It binds and neutralizes bacterial lipopolysaccharide and thereby abolishes the bioactivity of these toxic bacterial products [41,42,43,44,45]. TENP homologue of the emu has been also isolated from the egg white as a major protein, which exhibited antimicrobial activity to gram-positive bacteria, such as *Micrococcus luteus* and *Bacillus subtilis*, but not against gram-negative bacteria such as *Escherichia coli* and *Salmonella typhimurium* [35]. These results collectively suggest that this protein may have multiple functions as a component of BPI-like innate immune system during avian early embryonic development [29].

**Fig 7:**
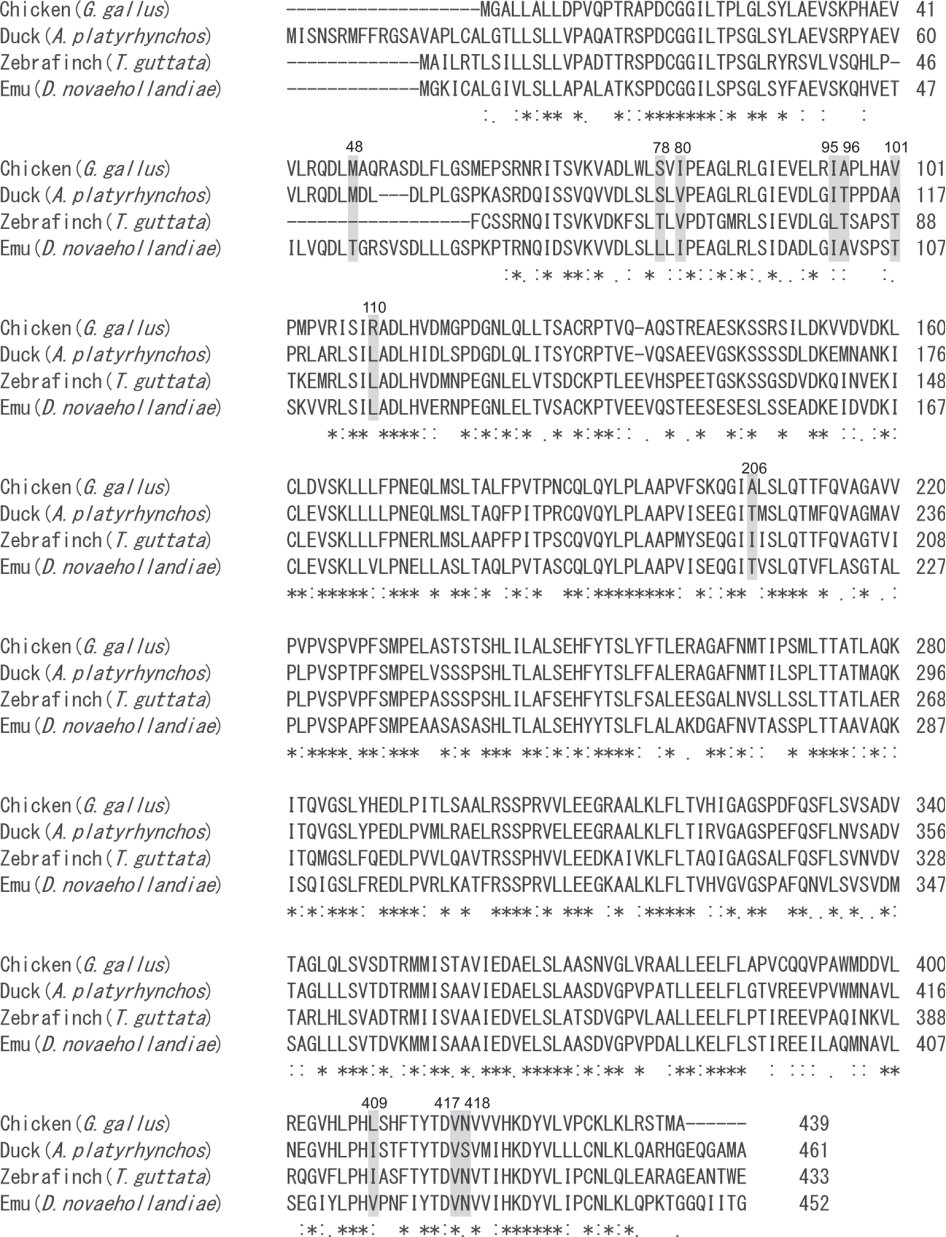
Comparison of the Amino Acid Sequences of TENP among Four Avian Species. Chicken (*Gallus gallus*) (AB219158), duck (*Anasplatyrhynchos*, Anserformes) (XP005011070), zebra finch (*Taeniopygia guttata*,Passeriformes) (XP012425675) and emu (*Dromaius novaehollandiae*, Struthioniformes) (AB556937). Asterisks indicate the amino acids that are common in all of the species. The positions of the amino acid substitutions detected in the chicken TENP are painted in gray.

The amino acid sequences of TENP are conserved among four avian species, including chicken (*Gallus gallus*, Phasianidae, Galliformes), duck (*Anas platyrhynchos*, Anserformes), zebra finch (*Taeniopygia guttata*, Passeriformes), and emu (*Dromaius novaehollandiae*, Struthioniformes); amino acid sequence identities, including gaps in the equivalent regions of the chicken G2 protein (439 aa) (Genbank accession no. AB219158), ranged from 72.0% (316/439) with *A*. *platyrhynchos* (XP_005011070) to 62.4% (274/439) with *D*. *novaehollandiae* (AB556937) and 59.2% (260/439) with *T*. *guttata* (XP_012425675). The amino acid substitutions had a tendency to be biased in the N-terminal region (Fig 7). Most of the amino acid substitutions detected in the chicken were not located in highly conserved regions. G2 protein has no N-terminal modification and seems to be less stable in egg white; therefore, amino acid substitution is involved in the egg white stability and/or, the defense against bacteria, rather than alteration in essential functions of the protein, as has been suggested in previous studies [46]. Our previous studies have shown that a non-synonymous substitution that results in a change in net charge on the protein is responsible for electrophoretic polymorphism in both chicken ovalbumin (OV) and in Japanese quail lysozyme (LYZ) [22,47]. In this study, we discovered a non-synonymous substitution (A329G), leading to Arg110Gln in the TENP protein, which uniquely discriminates G2^A^ from G2^B^ by the difference of electrophoretic mobility. Generally, the net charge of proteins depends on their amino acid composition as well as post-translational modifications such as addition of sialic acids and phosphate groups. The Arg110Gln substitution was expected to reduce from the total positive charge on G2^A^ by one unit due to the substitution of the positively charged hydrophilic polar amino acid (Arg) for a non-charged hydrophilic polar amino acid (Gln) at residue 110; thus, the electrophoretic mobility on the native-gel became faster than that of the G2^B^. However, the other ten amino acid substitutions found (refer to Table 3) are electrically neutral and as such probably do not affect the net charge on G2, and so we expect that they will not cause a difference in G2 electrophoretic mobility.

The *G2*^*B*^ allele was found to be dominant in almost all of the chicken breeds surveyed in this study. The *G2*^*A*^ allele was found in Japanese native breeds (namely, Ehime-Jidori, Chabo, Chahan, and Ukkokei), and was also found in two commercial layers (white and brown egg). In general, amino acid residues have different functional roles in the structure of a protein, such as in its activity, stability, and folding. Thus, amino acid substitutions found in this study may influence the function of ovoglobulin G2. However, the effects of these substitutions on biochemical properties of the G2 protein remain unknown. There have been several reports suggesting a correlation between variations in the G2 protein and economic traits such as egg production, egg weight, shell thickness, hatchability, etc. [48,49,50,51,52] as well as the embryonic fatality rate [49]. In addition, ovoglobulins have also been reported to be important in egg white foaming quality and viscosity [9,10], although their biological and food chemical functions have not been clearly elucidated. The availability of a wide range of chicken resources makes it potentially easy to identify novel mutations in the G2 protein gene and this could be of potential importance in establishing a relationship between G2 protein variants and favorable economic traits, such as resistance to disease and parasites, egg white and shell quality, and hatchability, all of which are of great potential importance to future poultry production. Further studies examining the correlation of *TENP* haplotype with various egg white traits using genetically diverse chicken resources are needed to develop a better understanding of the physiological functions of TENP in the chicken.

## Supporting Information

S1 Table Missense Mutations Found in the Coding Region of *TENP* cDNA Prepared from Seventy Eight Individual Samples Representing 28 Different Chicken Breeds, Lines, and Populations. cDNAs were prepared from RNA isolated from 21 chicken oviduct samples (representing six chicken breeds, lines, and populations) and their nucleotide sequences were determined. cDNAs were also prepared from 57 different embryos (representing 23 chicken breeds, lines, and populations) and their respective nucleotide sequences were also determined. Y, cytosine or thymine; R, adenine or guanine; M, adenine or cytosine.
(TIF)
