# Supplementary figures and images for: Identification of TENP as the Gene Encoding Chicken Egg White Ovoglobulin G2 and Demonstration of Its High Genetic Variability in Chickens
Source: PLoS One. 2016 Jul 29;11(7):e0159571. doi: 10.1371/journal.pone.0159571 (PMC4966965; doi:10.1371/journal.pone.0159571)

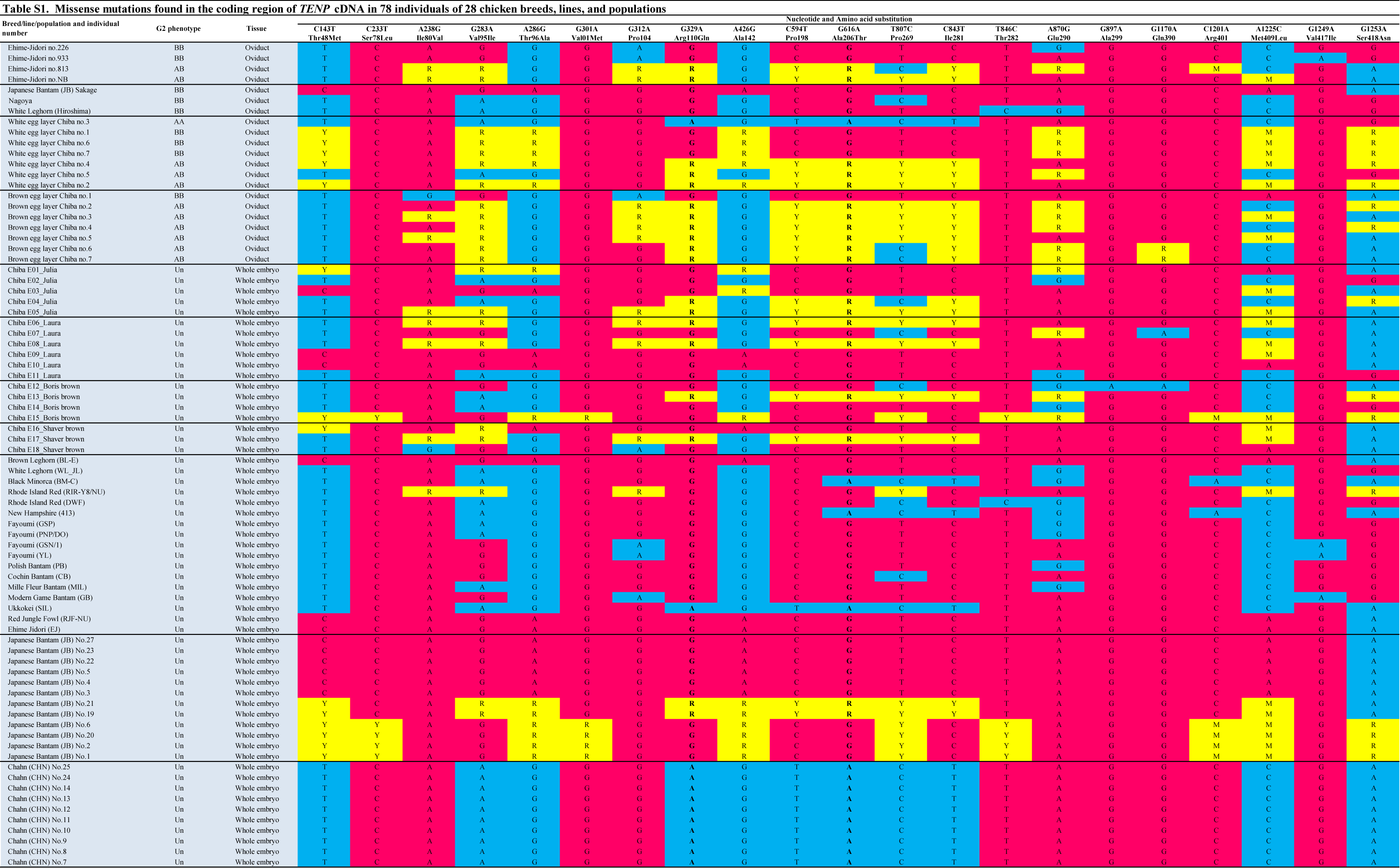

Supplement: S1 Table — cDNAs were prepared from RNA isolated from 21 chicken oviduct samples (representing six chicken breeds, lines, and populations) and their nucleotide sequences were determined. cDNAs were also prepared from 57 different embryos (representing 23 chicken breeds, lines, and populations) and their respective nucleotide sequences were also determined. Y, cytosine or thymine; R, adenine or guanine; M, adenine or cytosine. (TIF) [file pone.0159571.s001.tif]
